# Risk factors for early-onset venous thromboembolism in pediatric inflammatory bowel disease

**DOI:** 10.1093/crocol/otag034

**Published:** 2026-06-24

**Authors:** Caroline Chinchilla Putzeys, Nina Gautam, Andrew Ritchey, Amanda Kimberly, Umesh Sharma, Lucia Mirea, Laura Hamant, Hilary K Michel, Elizabeth Hilow, Ashish Patel, Sabina Ali, Jeremy Adler, Jonathan Moses, Brad Pasternak

**Affiliations:** Phoenix Children’s, Phoenix, AZ, United States; University of Arizona College of Medicine, Phoenix, AZ, United States; Phoenix Children’s, Phoenix, AZ, United States; University of Michigan, Ann Arbor, MI, United States; Mayo Clinic, Phoenix, AZ, United States; Phoenix Children’s, Phoenix, AZ, United States; Nationwide Children’s Hospital and The Ohio State University College of Medicine, Columbus, OH, United States; Nationwide Children’s Hospital and The Ohio State University College of Medicine, Columbus, OH, United States; Phoenix Children’s, Phoenix, AZ, United States; Phoenix Children’s, Phoenix, AZ, United States; University of California San Francisco Benioff Children’s Hospitals, San Francisco, CA, United States; University of Michigan, Ann Arbor, MI, United States; Stanford Medicine Children’s Health, Palo Alto, CA, United States; Phoenix Children’s, Phoenix, AZ, United States

**Keywords:** IBD, Crohn’s, ulcerative colitis, VTE, pediatrics

## Abstract

**Background:**

Inflammatory bowel disease (IBD) can lead to a hypercoagulable state, particularly at initial diagnosis. Consequently, venous thromboembolism (VTE) represents a serious complication associated with IBD. For children, the risk of VTE near the time of IBD diagnosis has not yet been quantified, and guidance for VTE prophylaxis remains unclear.

**Methods:**

This multisite matched case-control study examined pediatric patients with a new diagnosis of IBD from 2019-2024. Cases were those who developed VTE ≤30 days prior or ≤90 days post IBD diagnosis. Controls were newly diagnosed IBD patients without VTE. Each case was matched to 2-6 controls based on IBD subtype and age at IBD diagnosis. Demographics, laboratory values, prothrombotic risk factors and treatments were compared between matched cases and controls using conditional logistic regression stratified, by case sets.

**Results:**

Study subjects included 15 cases and 52 controls, resulting in 70 matched pairs. Factors associated with early VTE included lower hemoglobin (*P* = .003), lower albumin (*P* = .002), corticosteroids use (*P* = .01), central line (*P* = .002), and any surgery ≤90 days post IBD diagnosis (*P* = .001). No association with VTE was detected for platelets, C-reactive protein, fecal calprotectin, family history of IBD or body mass index.

**Conclusions:**

Albumin, hemoglobin, steroid use, central line, and any surgery ≤90 days post IBD diagnosis were associated with early VTE. These risk factors may be used in a future prediction model to stratify newly diagnosed pediatric IBD patients by their risk for early VTE development.

Key Messages

- **What is already known?** Active inflammatory bowel disease (IBD) increases the risk of venous thromboembolism (VTE), especially in hospitalized patients.
- **What is new?** Hemoglobin, albumin, steroid use, central line, and any surgery ≤90 days post IBD diagnosis are associated with VTE development in pediatric newly diagnosed IBD patients. No association was detected for platelets, C-reactive protein, fecal calprotectin, family history of IBD or body mass index with VTE risk.
- **How can this study help patient care?** Standardized VTE risk stratification and prophylaxis for newly diagnosed IBD pediatric patients or those with active disease to prevent mortality and morbidity.

## Introduction

Inflammatory bowel disease (IBD), which includes ulcerative colitis (UC) and Crohn’s disease (CD), is a chronic condition characterized by inflammation of the digestive tract. In the United States, approximately 125 per 100 000 individuals are affected by pediatric IBD.^1^ While primarily impacting the intestinal tract, IBD can also cause serious complications beyond the digestive system,^2^ such as heightened risk of venous thromboembolism (VTE).^3^^,^^4^

VTE is a recognized complication of active IBD, with common presentations including cerebral venous sinus thrombosis, portal, splenic, and hepatic vein thromboses, pulmonary embolism, and acute stroke—particularly in individuals with a patent foramen ovale (PFO).^5^^,^^6^ VTE can lead to serious consequences, such as sepsis,^7^ stroke,^8^ and post-thrombotic syndrome,^9^ in addition to adverse effects related to treatment. Patients who develop VTE typically experience prolonged hospitalizations, increased rates of ICU admission, and higher overall mortality.^10–12^

Adult patients with IBD have a well-established two- to threefold increased risk of VTE compared with adults without IBD.^13^ VTE risk increases if patients possess additional risk factors such as severe IBD, colonic involvement, recent colectomy,^14^ parenteral nutrition administration, central venous access, thrombophilia, systemic corticosteroid use,^15^ contraceptive use, smoking, or a family/personal history of VTE.^16^ The elevated risk of VTE persists in the post-hospitalization and postoperative phases between 90 days up to one year,^5^^,^^17^ especially in patients with reduced mobility.^18^ Treating physicians may be unaware of this risk or hesitant to provide VTE prophylaxis to adult patients experiencing acute IBD exacerbation due to concerns about possible increased bleeding risk, despite practice guidelines recommending pharmacologic prophylaxis for inpatient IBD patients.^19–21^

While the absolute risk of VTE is lower in children compared to adults, children with IBD have a significantly higher VTE risk compared to children without IBD.^22^ A recent meta-analysis revealed that children with IBD are two to three times more likely to develop VTE compared to children without IBD.^23^ This risk increases up to six-fold during hospitalization for active disease, likely driven by acute inflammation and the release of prothrombotic mediators.^24^ Pediatric studies have also demonstrated a higher relative risk of VTE in younger children (ages 0-8 and 9-11 years) compared with adolescents (ages 15-17 years) and adults, suggesting that age at IBD diagnosis is a risk factor for VTE formation.^5^^,^^24^ Additionally, some studies have identified major surgery, central venous catheter and steroid use as risk factors for VTE in pediatrics, though findings are not as well-established as in adults. A recent study also identified the period of highest absolute risk for VTE in children as six months prior to IBD diagnosis and persisting for up to one year thereafter, similar to adults.^5^

Currently, no evidence-based guidelines exist for VTE prophylaxis in pediatric inpatients with IBD. Despite this, in various healthcare institutions standard inpatient practices involve administering anticoagulation using low molecular weight heparin (LMWH) and/or using sequential compression devices (SCD) for patients hospitalized with severe IBD who have additional risk factors, as previously described.^25^ In the outpatient setting, standardized practices for VTE prophylaxis in pediatric IBD populations are currently lacking. This is critical as VTE risk in children remains elevated for a significant amount of time after IBD diagnosis.^5^ Therefore, it is crucial to quantify VTE risk in newly diagnosed pediatric patients with IBD to establish risk-stratified VTE prophylaxis guidelines for both inpatient and outpatient care.

There is a significant gap in management knowledge, requiring clear risk assessments and treatment guidelines for children newly diagnosed with IBD. Given these knowledge gaps, this study aimed to examine association of likely risk factors, and identify predictors for early VTE development in pediatric patients with IBD, that could be used for future risk-based, individualized treatment guidelines.

## Materials and methods

A retrospective matched case-control study was conducted across five tertiary pediatric centers in the United States. Prior to study initiation, all sites received Institutional Review Board (IRB) approval at their local institutions.

Pediatric patients aged 0-18 years with newly diagnosed IBD between 2019 and 2024 at participating sites were eligible for inclusion in this study. Cases included patients who developed VTE ≤30 days prior or ≤90 days post IBD diagnosis. Controls were selected from the same population of patients newly diagnosed with IBD who did not develop VTE in this timeframe. New IBD diagnosis was defined as the date of initial endoscopic and histologic confirmation documented in the medical record. Each case was matched to 2-6 controls based on IBD subtype (CD or UC) and age at diagnosis (±2 years). Demographics and baseline characteristics (age at diagnosis, sex, race, ethnicity, and body mass index at diagnosis) and IBD subtype were extracted from the medical record.

Laboratory values ≤90 days post IBD diagnosis included hemoglobin, platelet count, C-reactive protein (CRP), albumin, and fecal calprotectin (if available). Information on the primary outcome of venous thromboembolism (VTE) events included thrombi location and timing relative (≤30 days prior or ≤90 days post) to IBD diagnosis.

Additional clinical variables included history of surgery (including bowel surgery) ≤90 days post IBD diagnosis (and before VTE), presence of a central venous catheter, use of hormonal contraceptive, personal or family history of hypercoagulable disorders, family history of VTE in first-degree relatives, and family history of IBD and subtype in first-degree relatives. Data on medical management of IBD, including use of second-line therapies, were also collected.

### Statistical analyses

Descriptive statistics were used to summarize the distribution of demographic, baseline characteristics, and clinical factors for all study subjects, and separately for the VTE cases and matched controls. Differences in continuous measures between case-control pairs were computed as the control value minus the case value. For categorical factors, the concordance between case-control pairs was summarized. Statistical significance was tested using conditional logistic regression, stratified for each case set to account for matching a single case to multiple controls. Additionally, a multivariable conditional logistic regression model was developed using a self-derived stepwise selection approach (retaining *P*-value <.05), while addressing multicollinearity by examining associations among predictors. Given the limited sample size, the multivariable model considered only the most clinically relevant predictors to avoid overfitting. The degree of association was quantified using hazard-ratio (HR) estimates along with 95% confidence intervals (95% CI). All statistical tests performed were 2-sided and significance was evaluated at the 5% level. Statistical analyses were performed using SAS (Statistical Analysis Software 9.4; SAS Institute Inc.) or R software (version 4.1.2; R Foundation for Statistical Computing).

## Results

The total 67 study subjects included 15 VTE cases and 52 matched controls, resulting in 70 case-control pairs. Table 1 summarizes the distribution of baseline characteristics overall and within cases and controls separately. The majority of participants were documented as White (71.6%, *N* = 48), followed by Asian (11.9%, *N* = 8), Hispanic (10.4%, *N* = 7), and Black (6.0%, *N* = 4). Most subjects were of non-Hispanic ethnicity (88.1%, *N* = 59). The sex distribution was approximately equal among cases and controls. Overall, the mean (SD) of body mass index (BMI) at diagnosis was 19.75 (6.69) kg/m^2^ (Table 1). For CD patients, characteristics relating to the Paris classification are provided in Supplemental Table S1.

**Table 1:** Demographics and baseline characteristics of VTE cases and matched controls.

	Total (*N* = 67)	Controls (*N* = 52)	Cases (*N* = 15)
**Institution**			
** Site A**	12 (17.9%)	10 (19.2%)	2 (13.3%)
** Site B**	6 (9.0%)	5 (9.6%)	1 (6.7%)
** Site C**	31 (46.3%)	22 (42.3%)	9 (60.0%)
** Site D**	6 (9.0%)	5 (9.6%)	1 (6.7%)
** Site E**	12 (17.9%)	10 (19.2%)	2 (13.3%)
**Age**			
** Mean (SD)**	12.13 (3.38)	12.17 (3.35)	12.00 (3.61)
** Median**	13.00	13.00	13.00
** Range**	(3.00-18.00)	(3.00-18.00)	(4.00-18.00)
**Sex**			
** Female**	34 (50.7%)	26 (50.0%)	8 (53.3%)
** Male**	33 (49.3%)	26 (50.0%)	7 (46.7%)
**Race**			
** African-American**	4 (6.0%)	3 (5.8%)	1 (6.7%)
** Asian**	8 (11.9%)	5 (9.6%)	3 (20.0%)
** Hispanic**	7 (10.4%)	5 (9.6%)	2 (13.3%)
** White**	48 (71.6%)	39 (75.0%)	9 (60.0%)
**Ethnicity**			
** Hispanic**	8 (11.9%)	6 (11.5%)	2 (13.3%)
** Non-Hispanic**	59 (88.1%)	46 (88.5%)	13 (86.7%)
**BMI**			
** Mean (SD)**	19.75 (6.69)	19.37 (6.62)	21.09 (6.96)
** Median**	18.25	17.80	21.10
** Range**	(12.50-53.70)	(12.60-53.70)	(12.50-38.10)
**IBD Type**			
** Crohn’s Disease**	28 (41.8%)	23 (44.2%)	5 (33.3%)
** Ulcerative Colitis**	39 (58.2%)	29 (55.8%)	10 (66.7%)

Abbreviations: UCSF = University of California San Francisco; BMI = body mass index; IBD = Inflammatory Bowel Disease.

VTE cases had mean (standard deviation [SD]) age of 12.0 (3.6) years, and included 10 patients (66.7%) diagnosed with UC and 5 patients (33.3%) with CD (Table 1). Table 2 provides detailed descriptions of VTE cases including IBD type, days between diagnosis and VTE, thrombus locations, anticoagulation use at diagnosis, and other prothrombotic factors. The median number of days after IBD diagnosis for VTE development was 2 days, with three cases occurring prior to IBD diagnosis (range: 29 to 4 days before IBD diagnosis). Additionally, nine cases developed a thrombus, despite being on low molecular weight heparin for VTE prophylaxis while inpatient. Nine cases had central lines, seven cases had surgery within the past 90 days (all of which were bowel surgeries), one case had a personal history of hemoglobin C trait, and three cases had a family history of a hypercoagulable disorder, VTE, or IBD. The average number of IBD medical therapies (including 5-ASAs, corticosteroids, immunomodulators, JAK inhibitors, S1P receptor modulators, biologics, and anti-IL 12/23/integrins) used for cases who developed VTE was 1.93. Of the patients with VTE, seven (41%) were serious or potentially life threatening; three of these cases developed stroke, and one developed portal venous thrombus.

**Table 2:** Description of cases who developed VTE ≤30 days prior or ≤90 days post IBD diagnosis.

Age	Sex	IBD type	Days between IBD diagnosis and VTE	Thrombus location	AC at time of VTE diagnosis	Central line at time of VTE diagnosis	Surgery ≤90 days post IBD diagnosis	Use of OCP at time of VTE diagnosis	Personal history of hypercoagulable disorder	Family history of hypercoagulable disorder, VTE or IBD	Medical treatment
**4**	F	UC	37	Right internal jugular vein, Left internal jugular vein, right subclavian vein, left subclavian vein, left axillary vein, right cephalic vein, left basilic vein	N	N	Y	N	N	N	Methylprednisolone, infliximab
**6**	M	UC	2	Right basilic vein	LMWH	Y	N	N	N	N	Infliximab, vedolizumab
**12**	F	UC	−25	Superior sagittal sinus, right transverse sinus, right sigmoid sinus, right internal jugular vein, portal vein, IVC	LMWH	Y	N	N	N	Inherited thrombophilia, Crohn’s disease	Mesalamine, Budesonide
**13**	M	UC	41	Right subclavian vein, right axillary vein, right cephalic vein, right basilic vein	LMWH	Y	Y	N	N	N	Methylprednisolone, adalimumab, tofacitinib
**13**	M	UC	14	Left axillary vein	LMWH	Y	Y	N	N	N	Methylprednisolone, Infliximab, vedolizumab
**13**	F	UC	0	Right cephalic vein, right antecubital vein	N	N	N	N	N	N	Balsalazide, Methylprednisolone, infliximab
**13**	F	UC	2	Bilateral thalamic infarction, cerebral venous thrombosis, internal cerebral vein, vein of gallen, inferior sagittal sinus, straight sinus, proximal transverse sinus	N	N	N	N	N	N	Infliximab
**14**	F	UC	2	Left transverse sinus, left sigmoid sinus, “unknown upper limb veins”	N	Y	Y	N	N	N	Adalimumab
**14**	F	UC	9	Left cephalic lobe	N	N	N	N	N	N	Mesalamine, Prednisone, Infliximab
**16**	F	UC	54	No location provided	LMWH	Y	Y	N	N	VTE	Infliximab, Methylprednisolone
**9**	M	CD	28	Right internal jugular vein, right cephalic vein	LMWH	Y	N	N	Hemoglobin C trait	Sickle cell trait	Infliximab
**10**	M	CD	2	“Antecubital fossa”	N	N	N	N	N	N	Adalimumab
**11**	M	CD	−29	Right common iliac vein	LMWH	N	N	N	N	N	Infliximab
**14**	M	CD	30	Right basilic vein	LMWH	Y	Y	N	N	N	Infliximab
**18**	F	CD	−4	No location provided	LMWH	Y	Y	N	N	N	Prednisone, Methylprednisolone, Infliximab

Abbreviations: VTE = Venous thromboembolism; IBD = inflammatory bowel disease; CD = Crohn’s Disease; UC = Ulcerative Colitis; AC = anticoagulation; LMWH = low molecular weight heparin (enoxaparin) (prophylactic dosing); OCP = oral contraceptive pill.

The hazard for VTE was reduced with higher hemoglobin (HR = 0.68; *P* = .003) and higher albumin (HR = 0.24; *P* = .002) levels (Table 3). VTE cases were also more likely to have received corticosteroids (HR = 5.51; *P* = .01), have a central venous catheter (HR = 11.84; *P* = .002), and undergone surgery ≤ 90 days post IBD diagnosis (HR = 15.17; *P* = .001) (Table 4).

**Table 3:** Distribution of clinical factors overall, in VTE cases and matched controls separately, the paired differences (control value minus case value) and association of factors with VTE from conditional logistic regression.

Lab value	Total (*N* = 67)	Controls (*N* = 52)	Cases (*N* = 15)	**Difference (control minus case)** ^a^ **(*N* = 70)**	Hazard ratio (95% CI)	***P*-Value** ^b^
**Hemoglobin (g/dL)**					0.68 (0.53, 0.88)	.003
** *N***	67	52	15	70		
** Mean (SD) **	10.10 (2.74)	10.74 (2.54)	7.89 (2.27)	2.47 (3.39)		
** Median **	10.50	11.05	8.30	2.70		
** Range **	(2.90, 16.70)	(4.70, 16.70)	(2.90, 10.50)	(−5.80, 10.00)		
**Platelets (1000/mcL)**					1.00 (1.00, 1.01)	.12
** *N***	67	52	15	70		
** Mean (SD) **	453.36 (176.83)	432.56 (147.79)	525.47 (246.30)	−79.70 (259.26)		
** Median **	414.00	398.50	458.00	−60.00		
** Range **	(72.00, 1083.00)	(209.00, 822.00)	(72.00, 1083.00)	(−874.00, 534.00)		
**CRP (mg/dL)** ^c^					1.05 (1.00, 1.10)	.06
** *N***	63	50	13	59		
** Mean (SD) **	7.02 (10.92)	5.47 (9.21)	12.97 (14.88)	−5.72 (15.51)		
** Median **	2.80	1.40	7.80	−3.20		
** Range **	(0.10, 48.00)	(0.10, 48.00)	(0.40, 46.60)	(−45.60, 40.00)		
**Albumin (g/dL)**					0.24 (0.10, 0.59)	.002
** *N***	67	52	15	70		
** Mean (SD) **	3.43 (0.95)	3.68 (0.82)	2.58 (0.88)	0.88 (1.14)		
** Median **	3.50	3.70	2.60	0.75		
** Range **	(1.40, 5.00)	(1.70, 5.00)	(1.40, 4.50)	(−1.70, 3.40)		
**BMI**					1.07 (0.97, 1.18)	.20
** *N***	67	52	15	70		
** Mean (SD) **	19.75 (6.69)	19.37 (6.62)	21.09 (6.96)	−2.37 (6.84)		
** Median **	18.25	17.80	21.10	−1.73		
** Range **	(12.50, 53.70)	(12.60, 53.70)	(12.50, 38.10)	(−19.05, 15.60)		
**Fecal calprotectin (mcg/g)** ^c^					1.00 (1.00, 1.00)	.63
** *N***	57	44	13	56		
** Mean (SD) **	1681 (1139)	1693 (1133)	1638 (1204)	−36.63 (1694)		
** Median **	1506	1544	1250	0.00		
** Range **	(0.36, 4370)	(23, 4370)	(0.36, 3482)	(−3459, 3710)		

Abbreviations: CRP = C-reactive protein; SD = standard deviation.

a Differences were calculated using controls minus cases.

b *P* value from conditional logistic regression stratified by case sets.

c Analyses employed multiple imputations (10 iterations) on the log transformation, performed using a joint model assuming normality, considering each matched case set modeled using random effects. Results from iterations were combined using Rubin’s Rules.

The multivariable conditional logistic regression model retained surgery ≤ 90 days post IBD diagnosis and presence of a central venous catheter as risk factors for VTE (Table 5).

**Table 4:** Agreement of factors between VTE cases and matched controls, and association of factors with VTE from conditional logistic regression.

Factor	Total (*N* = 67)	Controls (*N* = 52)	Cases (*N* = 15)	VTE Case/control pairs (*N* = 70)				Hazard ratio (95% CI)	***P*-Value** ^a^
				Yes/Yes	Yes/No	No/Yes	No/No		
**Central line, *N* (%)**									
** No**	42 (75%)	37 (88.1%)	5 (35.7%)	7 (13%)	34 (63%)	4 (7.4%)	9 (17%)	11.84 (2.45, 57.11)	.002
** Yes**	14 (25%)	5 (11.9%)	9 (64.3%)						
**(*missing data *= 11, *missing pairs *= 16)**									
**Any surgery ≤ 90 days post IBD diagnosis, *N* (%)**									
** No**	58 (87%)	50 (96.2%)	8 (53.3%)	0 (0%)	33 (47%)	2 (2.9%)	35 (50%)	15.17 (3.10, 74.23)	.001
** Yes**	9 (13%)	2 (3.8%)	7 (46.7%)						
**Bowel surgery outside of 90 days of IBD diagnosis, *N* (%)**									
** No**	62 (92%)	48 (92.3%)	14 (93.3%)	0 (0%)	3 (4.3%)	4 (5.7%)	63 (90%)	0.68 (0.06, 8.44)	.76
** Yes**	5 (7.5%)	4 (7.7%)	1 (6.7%)						
**IBD first-degree relative, *N* (%)**									
** No**	57 (86%)	44 (84.6%)	13 (92.9%)	2 (3.1%)	3 (4.6%)	7 (11%)	53 (82%)	0.56 (0.06, 4.91)	.60
** Yes**	9 (14%)	8 (15.4%)	1 (7.1%)						
**(*missing data *= 1, *missing pairs *= 5)**									
**Need for second line agent, *N* (%)**									
** No**	43 (64%)	35 (67.3%)	8 (53.3%)	14 (20%)	21 (30%)	12 (17%)	23 (33%)	1.42 (0.45, 4.47)	.55
** Yes**	24 (36%)	17 (32.7%)	7 (46.7%)						
**Systemic corticosteroids, *N* (%)**									
** No**	54 (81%)	45 (86.5%)	9 (60.0%)	1 (1.4%)	29 (41%)	6 (8.6%)	34 (49%)	5.51 (1.50, 20.23)	.01
** Yes**	13 (19%)	7 (13.5%)	6 (40.0%)						
**OCP Use, *N* (%)**									
** No**	62 (100%)	48 (100%)	14 (100%)	0 (0%)	0 (0%)	0 (0%)	65 (100%)	-	-
** Yes**	0 (0%)	0 (0%)	0 (0%)						
**(*missing data *= 5, *missing pairs *= 5)**									
**Known hypercoagulable disorder, *N* (%)**								–	–
** No**	59 (98%)	47 (100%)	12 (92.3%)	0 (0%)	3 (5.1%)	0 (0%)	56 (95%)	-	-
** Yes**	1 (1.7%)	0 (0%)	1 (7.7%)						
**(*missing data *= 7, *missing pairs *= 11)**									
**Hypercoagulable disorder first-degree relative, *N* (%)**									
** No**	65 (97%)	52 (100%)	13 (86.7%)	0 (0%)	8 (11%)	0 (0%)	62 (89%)	-	-
** Yes**	2 (3.0%)	0 (0%)	2 (13.3%)						
**VTE first-degree relative, *N* (%)**									
** No**	66 (98%)	52 (100%)	14 (93.3%)	0 (0%)	5 (7%)	0 (0%)	65 (93%)	-	-
** Yes**	1 (1.5%)	0 (0%)	1 (6.7%)						

a *P* value obtained from conditional logistic regression.

**Table 5:** Multivariable conditional logistic regression examining risk factors for VTE in cases and matched controls.

Factor	Hazard ratio (95% CI)	*P*-Value
**Any surgery ≤90 days post IBD diagnosis**	26.04 (2.09, 323.93)	.011
**Central line**	12.68 (1.38, 116.96)	.025

Abbreviation: CI = confidence interval.

## Discussion

Virchow’s triad of endothelial injury, hypercoagulability, and venous stasis is highly prevalent in patients with active IBD. Intestinal and systemic inflammation contribute to intravascular endothelial injury.^26^^,^^27^ Hypercoagulability occurs as a sequela of inflammatory cytokines that upregulate procoagulant factors (fibrinogen, factor VIII) and decrease natural anticoagulants (antithrombin, protein C/S), increase platelet count and reactivity, and impair fibrinolysis.^26^^,^^27^ Venous stasis is promoted by immobility during periods of active disease, hospitalization, and surgical intervention.^23^ Therefore, VTE poses a significant risk for pediatric patients with active IBD, especially around the time of diagnosis. Currently, no guidelines exist for prophylactic measures in the outpatient setting.

Our analyses of 15 patients who developed VTE within the window of 30 days prior to or 90 days following IBD diagnosis revealed that laboratory values can provide risk stratification for VTE development. Among markers of systemic inflammation, lower hemoglobin levels, and reduced albumin concentrations, were significantly associated with the development of VTE. Notably, no association was detected for fecal calprotectin, a stool biomarker^28^ commonly used to assess intestinal inflammation in IBD, with VTE risk. Based on the prothrombotic risk factors identified in cases of VTE in this study, our findings are consistent with previous literature reporting that presence of a central line and recent surgery,^15^ are risk factors for VTE development. However, our findings further suggest that patients should be further assessed using laboratory values of albumin and hemoglobin, to better characterize their risk of VTE in the outpatient setting following IBD diagnosis. Additionally, while Harvey *et al.* suggested the window of “6 months prior to and 1 year following IBD diagnosis” as “the highest absolute risk period for VTE,”^5^ our results within our study’s restricted timeframe suggest that the highest absolute risk period for VTE development is rather centered on a systemic inflammatory response.

LMWH is known to be a safe and effective prophylactic and treatment option for VTE.^29^ Of note, its effectiveness in reducing thromboses associated with CVCs appears to be lower in critically ill infants (<1)^30^ and in adults with cancer.^31^ Chemoprophylaxis for VTE in patients with newly diagnosed IBD should be initiated early. One study showed that the availability of clear guidelines improved prophylaxis and diagnosis rates.^32^ Further risk stratification of VTE formation in pediatric patients with newly-diagnosed IBD based on laboratory values, along with clear guidelines of optimal dosing of anticoagulation, may greatly reduce the rates of VTE in the time period following IBD diagnosis.

VTE in pediatric patients can lead to significant adverse events. In those with IBD, the frequency of severe outcomes is elevated.^5^^,^^6^ Identifying risk factors for VTE development in these patients, along with a thorough assessment of VTE risk at the time of IBD diagnosis and active disease, is crucial for determining which patients would benefit most from inpatient and outpatient prophylaxis. This study enhances our understanding of these risks, contributing to the expanding body of literature on VTE in pediatric patients with IBD. While the current study leveraged case-control matching to reduce confounding bias and enhance statistical power to detect risk factors for VTE in newly diagnosed IBD children, the study limitations include small case sample size, and retrospective chart review with data unavailable for severity of disease activity and other unmeasured possible risk factors. Future research would benefit from a prospective longitudinal study of comprehensive VTE risk factors in pediatric IBD patients, focused on developing individualized, risk-based prophylaxis and treatment guidelines to minimize the risk of VTE and its associated complications.

In conclusion, the risk of VTE is significantly elevated in children with IBD around the time of IBD diagnosis. Among pediatric patients newly diagnosed with IBD, serum markers of inflammation (albumin, hemoglobin), use of corticosteroids, presence of a central venous catheter, and recent surgery were each associated with increased risk of developing VTE. This evidence emphasizes the need for standardized guidelines for VTE prophylaxis among children newly diagnosed with IBD who are at increased risk to target early interventions.

## Supplementary Material

otag034_Supplementary_Data

## Data Availability

The data underlying this study are derived from retrospective review of pediatric patient medical records across participating institutions and contain protected health information. In accordance with institutional review board approvals and privacy regulations, these data are not publicly available. De-identified aggregate data supporting the findings of this study may be made available from the corresponding author upon reasonable request and with appropriate institutional approvals. No new publicly accessible datasets were generated for this study.

## References

[1] Kappelman MD , Brensinger C, Parlett LE, Hurtado-Lorenzo A, Lewis JD. Prevalence of pediatric inflammatory bowel disease in the United States: pooled estimates from three administrative claims data sources. Gastroenterology. 2025;168:980-982.e2. 10.1053/j.gastro.2024.11.004 39577811 PMC12018129

[2] Ott C , Schölmerich J. Extraintestinal manifestations and complications in IBD. Nat Rev Gastroenterol Hepatol. 2013;10:585-595. 10.1038/nrgastro.2013.117 23835489

[3] Bernstein CN , Nugent Z, Singh H. Persistently high rate of venous thromboembolic disease in inflammatory bowel disease: a population-based study. Am J Gastroenterol. 2021;116:1476-1484. 10.14309/ajg.0000000000001237 33767104

[4] Ding Z , Sherlock M, Chan AKC, Zachos M. Venous thromboembolism in pediatric inflammatory bowel disease: an 11-year population-based nested case–control study in Canada. Blood Coagul Fibrinolysis. 2022;33:449-456. 10.1097/MBC.0000000000001166 36409922

[5] Harvey PR , McNulty D, Coupland B, Kemos P, Croft NM, Trudgill NJ. The risk of venous thromboembolism in children with inflammatory bowel disease. Inflamm Bowel Dis. 2025;31:izae249. 10.1093/ibd/izae249 39540429

[6] Aardoom MA , Klomberg RCW, Kemos P, et al The incidence and characteristics of venous thromboembolisms in paediatric-onset inflammatory bowel disease: a prospective international cohort study based on the PIBD-SETQuality Safety Registry. J Crohns Colitis. 2022;16:695-707. 10.1093/ecco-jcc/jjab171 34599822 PMC9228884

[7] Yeh YT , Tsai SE, Chen YC, et al Deep venous thrombosis and risk of consequent sepsis event: a retrospective nationwide population-based cohort study. Int J Environ Res Public Health. 2021;18:7879. 10.3390/ijerph18157879 34360172 PMC8345651

[8] S⊘rensen HT , Horvath-Puho E, Pedersen L, Baron JA, Prandoni P. Venous thromboembolism and subsequent hospitalisation due to acute arterial cardiovascular events: a 20-year cohort study. Lancet. 2007;370:1773-1779. 10.1016/S0140-6736(07)61745-0 18037081

[9] Chopard R , Albertsen IE, Piazza G. Diagnosis and treatment of lower extremity venous thromboembolism: a review. JAMA. 2020;324:1765-1776. 10.1001/jama.2020.17272 33141212

[10] Papay P , Miehsler W, Tilg H, et al Clinical presentation of venous thromboembolism in inflammatory bowel disease. J Crohns Colitis. 2013;7:723-729. 10.1016/j.crohns.2012.10.008 23127785

[11] Rohani P , Taraghikhah N, Nasehi MM, Alimadadi H, Assadzadeh Aghdaei H. Cerebrovascular events in pediatric inflammatory bowel disease: a review of published cases. Pediatr Gastroenterol Hepatol Nutr. 2022;25:180-193. 10.5223/pghn.2022.25.3.180 35611378 PMC9110847

[12] Chien KA , Cooley V, Prishtina F, Grinspan ZM, Gerber LM, Kucine N. Health and financial burdens associated with venous thrombosis in hospitalized children with inflammatory bowel disease. J Pediatr Gastroenterol Nutr. 2021;72:748-751. 10.1097/MPG.0000000000003052 33616374

[13] Aksan F , Monzur F. S1094 comparing the risk of venous thromboembolism and pulmonary embolism in inflammatory bowel disease patients treated with different biologics: a propensity score-matched, retrospective cohort study. Am J Gastroenterol. 2024;119:S774-S775. 10.14309/01.ajg.0001033744.10287.43

[14] Kaplan GG , Lim A, Seow CH, et al Colectomy is a risk factor for venous thromboembolism in ulcerative colitis. World J Gastroenterol. 2015;21:1251-1260. 10.3748/wjg.v21.i4.1251 25632199 PMC4306170

[15] Higgins PDR , Skup M, Mulani PM, Lin J, Chao J. Increased risk of venous thromboembolic events with corticosteroid vs biologic therapy for inflammatory bowel disease. Clin Gastroenterol Hepatology 2015;13:316-321. 10.1016/j.cgh.2014.07.017 25038374

[16] McKie K , McLoughlin RJ, Hirsh MP, Cleary MA, Aidlen JT. Risk factors for venous thromboembolism in children and young adults with inflammatory bowel disease. J Surg Res. 2019;243:173-179. 10.1016/j.jss.2019.04.087 31181463

[17] McCurdy JD , Kuenzig ME, Smith G, et al Risk of venous thromboembolism after hospital discharge in patients with inflammatory bowel disease: a population-based study. Inflamm Bowel Dis. 2020;26:1761-1768. 10.1093/ibd/izaa002 31995204

[18] Faye AS , Wen T, Ananthakrishnan AN, et al Acute venous thromboembolism risk highest within 60 days after discharge from the hospital in patients with inflammatory bowel diseases. Clin Gastroenterol Hepatol. 2020;18:1133-1141.e3. 10.1016/j.cgh.2019.07.028 31336196 PMC6980437

[19] Faye AS , Hung KW, Cheng K, et al Minor hematochezia decreases use of venous thromboembolism prophylaxis in patients with inflammatory bowel disease. Inflamm Bowel Dis. 2020;26:1394-1400. 10.1093/ibd/izz269 31689354 PMC7534414

[20] Sbeit W , Kadah A, Shafrir A, et al Unawareness of thromboprophylaxis is associated with low venous thromboembolism occurrence in hospitalized patients with acute inflammatory bowel disease flare. Minerva Med. 2020;111:560-565. 10.23736/S0026-4806.20.06885-8 32729705

[21] Tinsley A , Naymagon S, Enomoto LM, Hollenbeak CS, Sands BE, Ullman TA. Rates of pharmacologic venous thromboembolism prophylaxis in hospitalized patients with active ulcerative colitis: results from a tertiary care center. J Crohns Colitis. 2013;7:e635-e640. 10.1016/j.crohns.2013.05.002 23706933

[22] Mitchel EB , Rosenbaum S, Gaeta C, et al Venous thromboembolism in pediatric inflammatory bowel disease: a case-control study. J Pediatr Gastroenterol Nutr. 2021;72:742-747. 10.1097/MPG.0000000000003078 33605670 PMC9066981

[23] Zhang XY , Dong HC, Wang WF, Zhang Y. Risk of venous thromboembolism in children and adolescents with inﬂammatory bowel disease: a systematic review and meta-analysis. World J Gastroenterol. 2022;28:1705-1717. 10.3748/wjg.v28.i16.1705 35581968 PMC9048785

[24] Kappelman MD , Horvath-Puho E, Sandler RS, et al Thromboembolic risk among danish children and adults with inflammatory bowel diseases: a population-based nationwide study. Gut. 2011;60:937-943. 10.1136/gut.2010.228585 21339206

[25] Chien KA , Hammad HT, Gerber L, Sheth S, Sockolow R, Kucine N. Pediatric gastroenterologists’ approach to venous thromboembolism prophylaxis in pediatric inflammatory bowel disease. J Pediatr Gastroenterol Nutr. 2018;66:286-288. 10.1097/MPG.0000000000001690 28742719 PMC6612280

[26] Koutroubakis IE. The relationship between coagulation state and inflammatory bowel disease: current understanding and clinical implications. Expert Rev Clin Immunol. 2015;11:479-488. 10.1586/1744666X.2015.1019475 25719625

[27] Magro F , Soares J-B, Fernandes D. Venous thrombosis and prothrombotic factors in inflammatory bowel disease. World J Gastroenterol. 2014;20:4857-4872. 10.3748/wjg.v20.i17.4857 24803797 PMC4009517

[28] Bjarnason I. The use of fecal calprotectin in inflammatory bowel disease. Gastroenterol Hepatol (N Y). 2017;13:53-56. 28420947 PMC5390326

[29] Story E , Bijelic V, Penney C, Benchimol EI, Halton J, Mack DR. Safety of venous thromboprophylaxis with low‐molecular‐weight heparin in children with ulcerative colitis. J Pediatr Gastroenterol Nutr. 2021;73:604-609. 10.1097/MPG.0000000000003231 34676833

[30] Faustino EVS , Raffini LJ, Hanson SJ, et al Age-dependent heterogeneity in the efficacy of prophylaxis with enoxaparin against Catheter-Associated thrombosis in critically ill children: a post hoc analysis of a Bayesian phase 2b randomized clinical trial. Crit Care Med. 2021;49:e369-e380. 10.1097/CCM.0000000000004848 33566465 PMC7979442

[31] Kahale LA , Tsolakian IG, Hakoum MB, et al Anticoagulation for people with cancer and central venous catheters. Cochrane Database Syst Rev. 2018;6:CD006468. 10.1002/14651858.CD006468.pub6 29856471 PMC6389340

[32] Hamant LG , Gonzalez-Llanos L, Patel AS, et al Venous thromboembolism prophylaxis in pediatric inflammatory bowel disease patients hospitalized with a central line. J Pediatr Gastroenterol Nutr. 2023;76:610-615. 10.1097/MPG.0000000000003747 36821846

