# Supplementary material for: Risk factors for early-onset venous thromboembolism in pediatric inflammatory bowel disease
Source: Crohns Colitis 360. 2026 Jun 24;8(3):otag034. doi: 10.1093/crocol/otag034 (PMC13459966; doi:10.1093/crocol/otag034)
Supplement: Supplementary file 1 [file otag034_supplementary_data.docx]

**Table S1:** Characteristics of Crohn’s Disease Patients relating to the Paris Classification.

|  | **Total (N=28)** | **Controls (N=23)** | **Cases (N=5)** |
| --- | --- | --- | --- |
| **Location** |  |  |  |
| L1: distal 1/3 ileum (± limited cecal disease) | 6 (21.4%) | 5 (21.7%) | 1 (20.0%) |
| L2: colon | 7 (25.0%) | 6 (26.1%) | 1 (20.0%) |
| L3: ileocolon | 14 (50.0%) | 12 (52.2%) | 2 (40.0%) |
| L4a: upper GI disease proximal to the ligament of Treitz | 1 (3.6%) | 0 (0.0%) | 1 (20.0%) |
|  |  |  |  |
| **Behavior** |  |  |  |
| B1: non-stricturing, non-penetrating | 22 (78.6%) | 19 (82.6%) | 3 (60.0%) |
| B2: stricturing | 2 (7.1%) | 1 (4.3%) | 1 (20.0%) |
| B3: penetrating | 3 (10.7%) | 3 (13.0%) | 0 (0.0%) |
| B2B3: both stricturing and penetrating (at the same time or at different times) | 1 (3.6%) | 0 (0.0%) | 1 (20.0%) |
|  |  |  |  |
| **Perianal** |  |  |  |
| No | 24 (85.7%) | 21 (91.3%) | 3 (60.0%) |
| Yes | 4 (14.3%) | 2 (8.7%) | 2 (40.0%) |
|  |  |  |  |
| **Growth** |  |  |  |
| G0: no evidence of growth delay | 17 (60.7%) | 15 (65.2%) | 2 (40.0%) |
| G1: growth delay (at any time) | 11 (39.3%) | 8 (34.8%) | 3 (60.0%) |
